# Textbook of disaster psychiatry

**DOI:** 10.1080/20008198.2018.1468711

**Published:** 2018-05-03

**Authors:** Per-Olof Michel

**Affiliations:** Central Hospital, Karlstad, Sweden

## 1. Review

The editors are among the most respected pioneers in this field of science and inspired many of us who entered this field of knowledge in the late 1980s. The names that make up the advisory board is also a representative part of the most highly respected scholars. Even the list of co-authors contains an impressive list of individuals who present to the readers summaries of their area of interests. This background makes up a potentially good foundation for adding value into the field.

## 2. Content

The textbook is made up of five Parts and 22 Chapters, framed by the editors with an *Introduction* in Part I (Chapter 1), and closed with Part V, *Public Health and Disaster Psychiatry* (Chapter 22). It covers 351 pages and is filled with tables and figures to summarize and visualize important parts of the content. Every chapter is supported by important and relevant references regarding the area that is presented. The aim of this textbook, as stated in the Preface, is to provide ‘an updated, comprehensive review of the psychological, biological, and social processes of preparing, responding, and intervening for disaster mental health needs’.

The editors *Introduction* (Chapter 1) is an exposé that highlights what is to come. Part II, *Foundations of Disaster Psychiatry* (Chapters 2–5) is made up of five chapters that cover epidemiology, disaster ecology, neurobiology and different health trajectories. In Part III, *Clinical Care and Interventions* (Chapters 6–9) are narrated through four chapters that describe important aspects such as: early interventions, posttraumatic disorders, psychiatric aspects of medical-surgical disaster care and collaborative care for injured survivors. The concentration points of the textbook, that almost make up half of the volume, are placed in Part IV that contains *Special Topics* (Chapters 10–21). In these 11 chapters, light is shed on subjects such as: international disaster response, risk communication, consequences of media coverage, terrorism and weapons of mass destruction, children and families, disaster workers, health care planning, workplace disasters, pandemics, leadership, nuclear disasters responses and ethical issues. In the closing chapter (Chapter 22), *Public Health and Disaster Mental Health*, the editors sum up the preparation, responding and recovery aspects of disasters.

## 3. Added value

The textbook reaches its aforesaid aim and features important implications for public planning, when it comes to individual and community mental health care following disasters and terrorism. Beyond the important aspects of the foundations of disaster responses, preparedness, interventions and treatments, as well as resilience and recovery, the book also takes on other persisting issues. Examples that are underscored are risk communication, the challenges with media coverage of disasters, the vulnerability and resilience in children and families, workplace disasters, dangerous pandemics and nuclear disasters, as well as leadership in disasters and the importance of ethical considerations, especially during preparation phases.

## 4. Critical notes

There are some aspects that could have made the textbook even better. One of them is the title: ‘Disaster Psychiatry’. It may be argued that only a minor part of disasters, even if very important, will engage psychiatric aspects of wellbeing and health; most of preparedness, early interventions, responses, resilience and recovery in individuals and communities will lay in the hands of other societal authorities. Of course, experts in disaster psychiatry will still have important roles to fill in preparedness phases and when it comes to facilitating mental health in the population. Since the scene is so diverse, maybe the more inclusive construct of ‘Psychotraumatology’ could be more representative.

Another part is that the textbook is heavily balanced towards the US. Almost three-quarters of the authors have their affiliation in US, and a lot of examples of disasters, terrorist actions and school shootings are from there. There is also the sense that few of the presentations are descriptions of disasters from parts of the world where there is limited access to resources. This might of course reflect that US is heavily affected, and also that a lot of important research is done there.

Finally, it would have been good to see a chapter dealing with work related stress in civilian operational organizations, which make up a heavy part of the daily environment of rescue workers medical emergency services, police departments and other kinds of first responders in disaster settings. In such a chapter, important organizational and stress management aspects could have been described such as the first responder peer-support model Stress First Aid, which is mentioned only briefly (p. 94).

## 5. Summary

Reading the framing chapters gives the reader a sense of the editors aggregated involvement and commitment to the field. The textbook is not only for those considered by others as experts in the field, but also for societal authorities planning, preparedness and response authorities. Despite some critical aspects, the textbook inspires us to learn more about the ecology of disasters, the psychiatric aspects of medical-surgical disaster care, international disaster responses and especially about pandemics, work place disasters, nuclear accidents and ethical issues. This comprehensive review will definitely lead to important improvements with regard to nations preparedness, responses, interventions and treatment and for resilience and recovery in the face of disasters and terrorist actions. This textbook is highly recommended to those interested in the field of psychotraumatology.

